# Caesarean section rates in women in the Republic of Ireland who chose to attend their obstetrician privately: a retrospective observational study

**DOI:** 10.1186/s12884-020-03199-x

**Published:** 2020-09-21

**Authors:** Michael J. Turner, Ciara M. E. Reynolds, Léan E. McMahon, Eimer G. O’Malley, Michael P. O’Connell, Sharon R. Sheehan

**Affiliations:** UCD Centre for Human Reproduction, Coombe Women and Infants University Hospital, Cork Street, Dublin 8, Ireland

**Keywords:** Private health insurance, Packages of maternity care, Caesarean section, Clinical risk factors

## Abstract

**Background:**

Caesarean section (CS) rates are increasing and there are wide variations in rates internationally and nationally. There is evidence that women who attend their obstetrician privately have a higher incidence of CS than those who attend publicly. The purpose of this observational study was to further investigate why CS rates may be higher in women who chose to attend their obstetrician privately.

**Methods:**

This study analysed data collected as part of the clinical records by midwives at the woman’s first antenatal appointment in a large European maternity hospital. All women who delivered between the years 2009 and 2017 were included. Data were analysed both cross-sectionally and longitudinally.

**Results:**

Overall, 73,266 women had a singleton pregnancy and 1830 had a multiple pregnancy. Of the packages of maternity care, 75.2% chose public, 10.8% chose semiprivate and 14.0% chose private. During the study, 11,991 women attended the hospital for their first and second pregnancies. Overall, women who attended privately were older and had higher proportions of infertility treatment and history of miscarriage (all *p* < 0.001) compared to those publicly-funded. Private patients were more likely to have a history of infertility, a history of miscarriage, a multiple pregnancy and to be ≥35 yrs. They had lower rates of obesity, smoking and illicit drug use in pregnancy (all *p* < 0.001). In women who chose private care, the overall rate of CS was higher compared to women choosing publicly-funded (42.7% vs 25.3%, *p* < 0.001) The increase was due to an increase in elective rather than emergency CS. The increase in elective CS fell after adjustment for clinical risks. In the longitudinal analysis, 89.7% chose the same package second time around. Women who changed from public to private care for the second pregnancy were more likely to have had a previous emergency CS or admission to the Neonatal Unit.

**Conclusions:**

This study suggests that the increased CS rate in women privately insured may be attributed, in part, to the fact that women who can afford health insurance choose continuity of care from a senior obstetrician because they are risk adverse and wish to have the option of an elective CS.

## Background

A feature of childbirth in developed countries since the middle of the last century has been the escalation in caesarean section (CS) rates, with no evidence yet that they have plateaued [1–3]. There is also evidence of wide variations in CS rates internationally, nationally and locally [4, 5].

There are several reasons for the escalation and for the variations in CS. The operation has become much safer for mothers [6, 7]. Sociodemographic changes have increased the levels of risk factors such as advancing maternal age, rising obesity levels and the associated increased rates of gestational diabetes mellitus [8, 9]. Technical advances, such as ultrasound scanning, can identify cases where a CS is deemed to be in the fetal interest. Variations in CS rates may occur due to differences in risk factors in populations or differences in clinical practices. Finally, women and their obstetricians have become more averse to any perceived increase in fetal or maternal risk associated with vaginal delivery.

It has previously been reported that women whose maternity care is covered by private insurance have a higher CS rate than women whose care is publicly funded [10–12]. This higher CS rate has not been fully explained and has been reported in countries with different healthcare systems. The purpose of this observational study was to further investigate why CS rates may be higher in women who chose to attend their obstetrician privately.

## Methods

This retrospective observational study was conducted at the Coombe Women and Infants University Hospital in Dublin, Ireland’s capital city. The hospital is one of the largest maternity units in Europe and delivers annually approximately one in eight babies nationally. It accepts women from all socioeconomic backgrounds, from rural and urban settings, and offers private and public packages of maternity care. Thus, the population of the hospital is broadly representative of the national obstetric population [13, 14].

The data used were routinely collected and computerised by trained midwives as part of medical records. Data were first collected at the woman’s first prenatal appointment and updated following delivery and before discharge from the hospital with pregnancy outcome data. The system used to collect the data has standardised questions with a barcode system.

The study included all women who delivered a baby weighing > 499 g during the nine years 2009 and 2017. The data collected at the first antenatal visit included age, parity, employment status, marital status, pregnancy intention, nativity, psychiatric history, psychiatric medications, previous clinical data such as miscarriage history, and lifestyle data including current maternal smoking status. Height and weight were measured by the midwife at the first visit and recorded to one decimal place before Body Mass Index (BMI) was calculated.

The variables of interest in this study were the maternity package of care, which was categorised as ‘private’, ‘semi-private’ and ‘public’, and the mode of delivery which was coded as ‘elective caesarean’, ‘emergency caesarean’ and ‘vaginal delivery’. All nonplanned surgeries were termed ‘emergency’ and all planned surgeries were terms ‘elective’. In terms of package of care, in Ireland, maternity care is publicly funded by government for all women unless they choose a semi-private or private package. Public care is led by a senior obstetrician and responsibility is shared by a team of doctors, midwives, other healthcare professionals and general practitioners. Any inpatient care antenatally or in the postnatal period is provided on the public ward. Midwives and student midwives provide care to the woman during labour and birth with obstetric supervision provided by a trainee obstetrician and a consultant on call for emergencies.

Semi-private care differs in that antenatal and postnatal care may be provided in a private room subject to availability. The patient is supervised antenatally by the same senior obstetrician in the interests of continuity of care. In labour, the care is the same as that provided to a public patient.

Private care is a consultant-led package where the women choose to attend the same consultant obstetrician in their private consulting rooms on an ongoing basis throughout their pregnancy and in the postnatal period. Inpatient care is provided in a private room subject to availability. Per diem payments are required for hospital accommodation and these accommodation charges for semiprivate and private packages are covered by the women's health insurance company. In the private package, the obstetrician’s fees are paid for by the woman personally. The insurance company may contribute a small co-payment. Irrespective of the package of care, all women are delivered in the same labour ward, in single ensuite rooms, or operating theatre and are cared for by the same staff.

All data were analysed using statistical software programme SPSS version 24.0 and the Vassarstats online statistical programme [15]. Normality of data was assessed by the skewness and kurtosis values, examination of histograms and the Kolmogorov Smirnov statistics. All normally distributed data were reported as means and standard deviations (SD) and all non-parametric data were reported as medians and interquartile ranges (IQR). Table 1 and supplementary tables 1, 2 and 3 were analysed using descriptive statistics. Differences between variables were assessed using the test for significance of the difference between two independent proportions test using the calculation of z-ratios or one-way analysis of variance (ANOVA) for difference in mean values. Tables 2 and 4 and supplementary table 4 were analysed using the descriptive statistic crosstabulation analysis. Table 3 and supplementary table 5 were analysed using logistic regression models. Confounding variables were chosen based on either their significant association with the outcome variables on univariate analysis or their previously suggested associations in the literature. The study was approved by the Coombe Women and Infants University Hospital Research Ethics Committee (4–2013).

**Table 1:** Characteristics of women with singleton pregnancies by package of care

	*n*	Total	Public	Semi-private	Private
		*n* = 73,266	*n* = 55,072	*n =* 7905	*n* = 10,289
Age (years; mean, SD)	73,266	31.3 (5.6)	30.2 (5.6)	33.9 (3.8)	35.5 (3.8)^a^
Age < 35 years (%)	50,925	69.5	77.1	55.7	39.7^a^
Age 35–39 years (%)	18,369	25.1	19.1	38.4	46.7^a^
Age ≥ 40 years (%)	3972	5.4	3.8	5.9	13.6^a^
Elective CS (%)	10,257	14.0	11.1	14.1	29.4^a^
Emergency CS (%)	10,280	14.0	14.2	14.1	13.3^b^
Vaginal delivery (%)	52,708	72.0	74.7	71.8	57.3^a^
Nulliparas (%)	29,376	40.1	40.7	41.5	35.9^a^
Married/Civil Partnership (%)	47,083	64.3	55.9	86.5	92.1^a^
Irish-born (%)	51,371	70.3	63.5	90.3	91.1^a^
Infertility treatment (%)	2649	3.6	2.1	4.5	11.3^a^
Planned pregnancy (%)	48,667	66.5	61.4	82.2	81.7^a^
BMI (median, IQR)	72,718	24.5 (6.0)	24.7 (6.3)	24.4 (5.1)	23.8 (4.8)^a^
Underweight (%)	1931	2.6	2.5	1.0	4.8^a^
Normal weight (%)	37,833	51.6	50.0	54.5	58.3^a^
Overweight (%)	21,198	28.9	29.1	31.3	26.2^a^
Obesity (%)	12,304	16.8	18.5	13.2	10.7^a^
Professional/managerial employment (%)	18,720	25.7	16.8	43.3	59.7^a^
Unemployed (%)	5559	7.6	10.0	0.9	0.5^a^
Current depression (%)	1192	1.6	2.0	0.7	0.5^a^
Current anxiety (%)	2704	3.7	4.1	3.0	1.9^a^
Anxiolytics/antidepressants (%)	1533	2.1	2.3	1.3	1.5^a^
Smoked in pregnancy (%)	9209	12.6	16.0	3.2	1.3^a^
Any alcohol use in pregnancy (%)	1114	1.5	1.5	2.1	1.3^c^
Illicit drugs in pregnancy (%)	1158	1.6	2.0	0.5	0.1^a^

Superscript letters denote differences between the columns ‘public’ and ‘private’

Significance: a = *p* < 0.001, b = *p* < 0.01, c = *p* < 0.05

**Table 2:** Characteristics of all women with elective caesarean section by age group and maternity package of care (based on medical insurance status)

Years		Medical Insurance Status			Total
		Private	Semi-private	Public	
< 18	n	0	2	436	438
	%	0.0%	0.0%	0.8%	0.6%
18–24	n	59	84	8853	8996
	%	0.6%	1.1%	16.1%	12.3%
25–29	n	483	811	14,724	16,018
	%	4.7%	10.3%	26.7%	21.9%
30–34	n	3543	3510	18,420	25,473
	%	34.4%	44.4%	33.4%	34.8%
35–39	n	4804	3034	10,531	18,369
	%	46.7%	38.4%	19.1%	25.1%
40	n	1400	464	2108	3972
	%	13.6%	5.9%	3.8%	5.4%
	n	10,289	7905	55,072	73,266

**Table 3:** Unadjusted and adjusted relationships between package of care and mode of delivery stratified by parity and age group

	Private	
	Unadjusted ORs (95% CI)	Adjusted ORs (95% CI)
Model 1: All women	*n* = 10,289	
Vaginal delivery	Reference	Reference
Elective caesarean	3.5 (3.3–3.6)^a^	2.9 (2.8–3.1)^a^
Emergency caesarean	1.2 (1.1–1.3)^a^	1.3 (1.2–1.4)^a^
Model 2: All Nulliparas	*n* = 3698	
Vaginal delivery	Reference	Reference
Elective caesarean	5.8 (5.2–6.4)^a^	4.4 (3.9–4.9)^a^
Emergency caesarean	1.3 (1.2–1.5)^a^	1.2 (1.0–1.4)^c^
Nulliparas ≥35 years		
Vaginal delivery	Reference	Reference
Elective caesarean	4.1 (3.5–4.8)^a^	3.8 (3.1–4.6)^a^
Emergency caesarean	1.1 (0.9–1.3)	1.2 (1.0–1.4)^c^
Nulliparas ≥40 years		
Vaginal delivery	Reference	Reference
Elective caesarean	5.1 (3.6–7.2)^a^	4.9 (3.3–7.3)^a^
Emergency caesarean	1.4 (0.9–1.9)	1.4 (0.9–2.1)
Model 3: All Multiparas	*n* = 6591	
Vaginal delivery	Reference	Reference
Elective caesarean	2.9 (1.2–1.4)^a^	3.0 (2.8–3.2)^a^
Emergency caesarean	1.2 (1.1–1.3)^a^	1.4 (1.3–1.6)^a^
Multiparas ≥35 years		
Vaginal delivery	Reference	Reference
Elective caesarean	2.4 (2.2–2.6)^a^	2.7 (2.5–2.9)^a^
Emergency caesarean	0.9 (0.8–1.1)	1.2 (1.0–1.4)^c^
Multiparas ≥40 years		
Vaginal delivery	Reference	Reference
Elective caesarean	2.7 (2.3–3.2)^a^	2.9 (2.4–3.6)^a^
Emergency caesarean	1.0 (0.8–1.3)	1.2 (0.9–1.8)

Overall reference category: Publicly funded; *n* = 55,072

Model 1: Adjusted for age > 35 years, BMI, History of infertility, History of miscarriage, nativity, and smoking status

Models 2 and 3: Adjusted for BMI, History of infertility, History of miscarriage, nativity, and smoking status

Significance: a = *p* < 0.001, b = *p* < 0.01, c = *p* < 0.05

**Table 4:** Mode of delivery in second pregnancy of women who were delivered by Caesarean section (CS) previously analysed by package of maternity care

	Public *n* = 1999	Semi-private *n* = 456	Private *n* = 759
	% (*n*)	% (*n*)	% (*n*)
Vaginal delivery	22.8 (456)^a^	24.1 (110)	10.1 (77)^a^
Emergency CS	21.4 (428)^a^	16.4 (75)	12.9 (98)^a^
Elective CS	55.8 (1115)^a^	59.4 (271)	76.9 (584)^a^

*P* values comparing private vs public

Significance: a = p < 0.001, b = p < 0.01, c = p < 0.05

## Results

Over the nine years there was a total of 75,096 cases eligible for inclusion in the study. Of these, 73,266 were singleton pregnancies and 1830 were multiple pregnancies. Overall, 75.2% chose a public package of care, 10.8% chose a semi-private and 14.0% chose a private package of care. In terms of mode of delivery, 14.5% delivered by elective CS, 14.5% also delivered by emergency CS and 71.0% delivered vaginally.

Table 1 shows the characteristics of the study population, excluding multiple births analysed according to whether the woman chose public, semi-private, or private care. Women who chose private care were on average 5.3 years older (*p* < 0.001), and were more likely to be Irish-born (*p* < 0.001), married (*p* < 0.001), and in professional/managerial employment (*p* < 0.001) compared to public patients. A higher proportion of women who chose the private package of care had been treated for infertility (*p* < 0.001), but they had higher rates of planned pregnancy (*p* < 0.001), lower proportions of obesity (*p* < 0.001), smoking (*p* < 0.001), and illicit drug use (*p* < 0.001) in pregnancy. Overall, the CS rate was higher at 42.7% for private patients, compared with 28.2% for semiprivate patients (*p* < 0.001) and 25.3% for public patients (*p* < 0.001).

The number of women opting for private care with a multiple pregnancy was 24.6% (*n* = 450) compared with 14.9% (*n* = 10,289) of women with a singleton pregnancy (Supplementary Table 1). Overall, in the nine years there were 1774 sets of twins, 54 sets of triplets and two sets of quadruplets. The demographic differences between women with a multiple pregnancy opting for private care compared with those opting for public care were similar to singleton pregnancies but more than half had a history of infertility treatment. The CS rate for all multiple pregnancies was high but women opting for private care had a higher proportion of elective CS (private vs. public; 53.9% vs 35.7%, *p* < 0.001 and private vs. semi private; 53.9% vs. 40.8%; *p* = 0.011) whereas those that did not were more likely to have an emergency CS (private vs. public; 30.3% vs. 47.2%, *p* < 0.001 and private vs. semi-private; 30.3% vs. 41.8%, *p* = 0.015).

Supplementary Table 2 shows the characteristics of the study population analysed for nulliparas and Supplementary Table 3 shows the characteristics of the study population analysed for multiparas. The overall CS rate was higher in private patients compared with public patients. However, with all three packages of care, the emergency CS rate was higher in nulliparas and the elective CS rate was higher in multiparas due to repeat elective CS in women with a previous CS (Supplementary Table 4).

Table 2 shows the age categorisation of women analysed according to the package of care chosen. Women opting for private care were more likely to be in the 35–40 years age group or in the > 40 years age group. Only 5.3% (*n* = 542) of private patients were < 30 years old.

The elective CS rate was 29.4% (*n* = 10,289) in private patients compared with 11.1% (*n* = 55,072) in public patients (*p* < 0.001) but the rate of emergency CS was lower (13.3% vs. 14.2%, *p* = 0.007). When adjusting for BMI, history of infertility or miscarriage, and nativity, the OR for elective CS in private patients was 2.5 (95% CI 2.3–2.6, *p* < 0.001) (Table 3). The adjusted OR for elective CS increased in both age categories ≥35 and ≥ 40 years, but the increase was greater in nulliparas compared with multiparas.

The study also analysed longitudinally the packages of care chosen over the nine years for a woman’s first delivery compared with her second (*n* = 11,990) (Table 4 and supplementary Tables 4 and 5). Overall, 89.7% of women chose the same package second time around. Of the 8503 women who chose a public package of care for the first delivery, 95.3% did so for the second. Of the 1578 women who chose a semiprivate package of care for the first delivery, 63.8% did so for the second. Of these, 450 changed to public care and there was a significant increase in their unemployment rate after their first delivery (*p* < 0.01). Of the 1908 women who chose a private package of care for the first delivery, 86.3% did so for the second.

Table 4 shows the modes of delivery in the second pregnancy of women who were delivered by CS in their first pregnancy, stratified by package of maternity care. Private patients in the second pregnancy had a higher rate of elective CS compared to public patients (76.9% vs. 55.8%, *p* < 0.001) however, they had lower rates of emergency CS second time around (12.9% vs. 21.4%, p < 0.001). Private patients also had a half the rate of vaginal delivery after CS (VBAC) compared to public patients (10.1% vs. 22.8%, *p* < 0.001).

Women who changed from private care to semiprivate care after their first delivery (*n* = 117) were less likely to have been delivered by elective CS previously (*p* < 0.01). Women who changed from private to public care (*n* = 144) were more likely to be younger (*p* < 0.01), to be unemployed (*p* < 0.01) and to have an unplanned pregnancy (*p* < 0.001).

Women who changed from public to private care (*n* = 155) were more likely to have been delivered by emergency CS for their first delivery and more likely to have a history of infertility treatment (*p* < 0.01) and a history of miscarriage (*p* < 0.01). Women who changed from public to semiprivate (*n* = 247) were also more likely to have been delivered by emergency CS for their first delivery (*p* < 0.05). The number of perinatal deaths in women who changed their package of care was too low for analysis.

## Discussion

This observational study in the capital city of a high-income country found that the CS rate was 17.4% higher in women who chose to attend their obstetrician privately compared with those that were completely funded publicly. There was a significant increased risk of elective CS in private patients rather than emergency CS compared with women who did not chose private care with their individual obstetrician.

Women who attended privately were more likely to have risk factors that increased their likelihood of CS such as advanced maternal age, and multiple pregnancy. Furthermore, women choosing private care were more likely to have had a previous pregnancy loss or a history of treatment for infertility. It is notable that assisted reproduction in Ireland is privately funded so it is common for women who conceive after infertility treatment to opt for private maternity care.

An important strength of this study is that all the sociodemographic and clinical data were computerised by a midwife as part of the medical records. Furthermore, to our knowledge, such data has not been previously linked longitudinally. The longitudinal analysis showed that few women change their package of care after their first delivery. Those that changed to private care were more likely to have had complications with the first delivery. Those that changed from private care were less likely to be in well paid employment.

A potential weakness is that it was based on a single centre study and the findings may not be generalisable in other settings. The advantage of this centre, however, was that private and public patients were managed in the same delivery suite, by the same doctors and midwives following the same local and national clinical guidelines. We also did not have details of maternal and neonatal comorbidities such as pre-gestational diabetes and hypertension, gestational diabetes and hypertension or fetal growth retardation during pregnancy to adjust for in analysis and the main indication for surgery could not be determined.

A weakness of the study is that we do not have high quality data on the women’s previous medical complications, for example cardiovascular disease, and there may be other confounders that influenced a woman’s choice of care or influenced the planned mode of delivery.

In a previous large Irish retrospective cohort study of 403,642 childbirth hospitalisations from 2005 to 2010, women with private care coverage were more likely to have an elective CS (RR 1.48; 95% CI 1.45–1.51) and were more likely to have an emergency CS (RR 1.25; 95% CI 1.22–1.27) [12]. This study used an administrative dataset based on hospitalisations and not a clinical dataset based on consecutive births. The authors acknowledged that, despite multivariable analysis, there could be important residual confounders, for example, maternal variables such as parity, obesity, assisted reproduction, ethnicity and socioeconomic group and fetal variables such as growth restriction, macrosomia, malposition [12]. The study also lacked information on multiple pregnancies, number of previous CS, and on women who changed between public and private care in different pregnancies.

In a study of 29,870 nulliparous, singleton births delivered in Ireland in 2009, the administrative dataset based on hospital discharges was linked with a clinical dataset from the National Perinatal Reporting System [16]. The overall CS rate was 26.1% and 79.6% were emergency CS. The CS rate was higher in women who chose private care but after adjustment for maternal, clinical and hospital characteristics, the rate was significantly increased for elective CS but not emergency CS. The study, however, was not able to adjust for other potentially important antenatal variables such as maternal obesity, assisted reproduction. Also, there was a lack of information on CS for maternal request. It may be that women choose private care to fulfil their own preference for an elective CS even in the absence of a clinical indication.

A recent Irish report examined the factors associated with the choice of maternity care pathway [10]. Increased maternal age, higher socioeconomic grouping of women, and birth in Ireland were all positively associated with choosing private care but the levels of risk identified in early pregnancy were not. Women in private care were more likely to be delivered by CS (RR 1.98, *p* < 0.01). However, this was a relatively small survey of 1789 nulliparas who participated in a prospective study on maternal morbidity postpartum [10]. Information was not available on the type of CS or on pre-pregnancy risk factors such as infertility. In a previous study from our hospital on maternal obesity trends and increasing CS rates between 2009 and 2014, the increase was found to be strongly associated with increasing maternal age, particularly in nulliparas [8].

In a small postnatal survey from 1995 to 1997 in Chile, the rate of elective CS was 30–68% in women with private obstetricians compared with 12–14% in women not attending a private obstetrician [17]. It was suggested that patient choice was unlikely to be the primary explanation, but no data were presented on clinical or sociodemographic variables.

In a systematic review and meta-analysis of women’s preferences for CS in Argentina, 38 studies (*n* = 19,403) were included [18]. A higher preference for CS was reported by women who had a previous CS compared with those women without a previous CS.

In an Australian single centre, retrospective, cross-sectional study from 2007 to 2014 of 61,355 singleton term deliveries, neonatal outcomes following emergency CS were worse for women choosing public care rather than private care [19]. The differences in the public deliveries in low Apgar scores at 5 min, admissions to the Neonatal Critical Care and respiratory distress all persisted after multivariable analysis for feto-maternal factors.

In a large retrospective analysis of hospital discharge data from the United States of nearly 7 million births in 2002–2009, the births covered by the Government’s Medicaid programme had a lower odds of CS (aOR 0.91) compared with privately insured births, even after controlling for clinical, demographic and hospital variables [11]. The authors acknowledged that the difference may be due to clinical and sociodemographic factors and provider factors not captured in the data. During the study, the CS rate also escalated at a faster rate in women who were privately insured.

We also found that women who chose the private package of care had half the rate of vaginal delivery second time around. Another Irish study found that the overall increase in CS rate in two large Irish maternity hospitals was associated with a major decrease in the incidence of VBAC [20]. Two previous studies found that duration since last birth, having had midwifery care during pregnancy, being advised to attempt a VBAC by their healthcare provider were more likely to choose ato trial a VBAC whereas those who were in the obesity category were less likely to trial a VBAC [21, 22].

## Conclusions

Our study shows the overall increase in the CS rate in women choosing private maternity care is significantly associated with an increase in elective CS, where the mode of delivery is agreed in partnership by the woman and her obstetrician antenatally. It shows, unlike previous studies, that women who chose the private package are more likely to have a history of previous pregnancy loss, infertility and multiple pregnancy and they are more likely to be > 35 years old. This suggests that women are choosing private care, in part, because they are more risk averse for clinical and sociodemographic reasons. They prefer a model of care where there is continuity of care by a senior obstetrician and where they believe they can optimise a good clinical outcome for their baby and themselves [23]. As the rate of emergency CS was not increased in women attending for private care, the findings indicate that payment for obstetricians to perform a CS is unlikely to be a factor in the overall increased CS rate in private patients within the Irish healthcare system. Our findings may not be applicable in other healthcare systems internationally. However, what our study does highlight is that the association between an increased CS rate and private health insurance is complex and that future investigations in any system needs to consider women’s choices and clinical variables in detail rather than simplistic fiscal considerations alone.

## Supplementary information

**Additional file 1: Supplementary Table 1.** Characteristics of women with multiple births by package of care.

**Additional file 2: Supplementary Table 2.** Characteristics of nulliparas by package of maternity care.

**Additional file 3: Supplementary Table 3.** Characteristics of multiparas by package of maternity care.

**Additional file 4: Supplementary Table 4.** Changes in mode of delivery between pregnancies by package of maternity care in second pregnancy (*n* = 11,990).

**Additional file 5: Supplementary Table 5.** Factors associated with changes in model of care between private and public.

## Data Availability

The data that support the findings of this study are available from the Coombe Women and Infants University Hospital but restrictions apply to the availability of these data, which were used under license for the current study, and so are not publicly available. Data are however available from the authors upon reasonable request and with permission of the Coombe Women and Infants University Hospital.

## Abbreviations

CS: Caesarean section
BMI: Body mass index
SD: Standard deviation
IQR: Interquartile range
ANOVA: Analysis of variance
