# Supplementary material for: Caesarean section rates in women in the Republic of Ireland who chose to attend their obstetrician privately: a retrospective observational study
Source: BMC Pregnancy Childbirth. 2020 Sep 21;20:548. doi: 10.1186/s12884-020-03199-x (PMC7504647; doi:10.1186/s12884-020-03199-x)
Supplement: Supplementary file 1 — Additional file 1: Supplementary Table 1. Characteristics of women with multiple births by package of care. [file 12884_2020_3199_MOESM1_ESM.docx]

Supplementary Table 1. Characteristics of women with multiple births by package of care.

|  |  | Total | Public | Semi-private | Private |
| --- | --- | --- | --- | --- | --- |
|  | *n* | *n*=1830 | *n*=1243 | *n=*137 | *n*=450 |
| Twin sets (%) | 1774 | 96.9 | 97.1 | 99.3 | 95.8 |
| Triplet sets (%) | 54 | 3.0 | 2.8 | 0.7 | 4.0 |
| Quadruplet sets (%) | 2 | 0.1 | 0.1 | 0.0 | 0.2 |
| Age (years; mean, SD) | 1830 | 33.3 (5.3) | 32.3 (5.4) | 34.2 (3.7) | 36.1 (4.3) |
| Age <35 years (%) | 1056 | 57.7 | 65.9 | 56.2 | 35.6 |
| Age 35-39 years (%) | 571 | 31.2 | 25.7 | 38.0 | 44.4 |
| Age ≥ 40 years (%) | 203 | 11.1 | 8.4 | 5.8 | 20.0 |
| Elective CS (%) | 533 | 41.2 | 35.7 | 40.8 | 53.9 |
| Emergency CS (%) | 545 | 42.1 | 47.2 | 41.8 | 30.3 |
| Vaginal delivery (%) | 217 | 16.8 | 17.1 | 17.3 | 15.8 |
| Nulliparas (%) | 833 | 45.5 | 43.1 | 51.1 | 50.4 |
| Married/Civil Partnership (%) | 1329 | 72.7 | 65.1 | 82.5 | 90.9 |
| Irish-born (%) | 1385 | 75.9 | 68.2 | 92.0 | 92.4 |
| Infertility treatment (%) | 544 | 29.7 | 21.0 | 30.7 | 53.6 |
| Planned pregnancy (%) | 945 | 51.7 | 54.2 | 60.6 | 42.0 |
| BMI (median, IQR) | 1830 | 25.0 (6.4) | 25.3 (7.0) | 24.6 (5.0) | 24.4 (5.7) |
| Underweight (%) | 24 | 1.3 | 1.6 | 0.0 | 0.9 |
| Normal weight (%) | 881 | 49.2 | 45.9 | 54.7 | 57.0 |
| Overweight (%) | 553 | 30.9 | 31.2 | 32.1 | 29.8 |
| Obesity (%) | 331 | 18.5 | 21.3 | 13.1 | 12.2 |
| Professional/managerial employment (%) | 578 | 32.1 | 22.0 | 39.1 | 57.5 |
| Unemployed (%) | 108 | 6.0 | 8.2 | 1.5 | 1.3 |
| Current depression (%) | 21 | 1.1 | 1.5 | 1.5 | 0.0 |
| Current anxiety (%) | 58 | 3.2 | 3.9 | 0.7 | 2.0 |
| Anxiolytics/antidepressants (%) | 32 | 1.7 | 1.9 | 0.7 | 1.8 |
| Smoked in pregnancy (%) | 191 | 10.4 | 13.8 | 6.6 | 2.2 |
| Any alcohol use in pregnancy (%) | 12 | 0.7 | 0.9 | 0.0 | 0.2 |
| Illicit drugs in pregnancy (%) | 12 | 0.7 | 0.9 | 0.0 | 0.2 |
