# Supplementary material for: Caesarean section rates in women in the Republic of Ireland who chose to attend their obstetrician privately: a retrospective observational study
Source: BMC Pregnancy Childbirth. 2020 Sep 21;20:548. doi: 10.1186/s12884-020-03199-x (PMC7504647; doi:10.1186/s12884-020-03199-x)
Supplement: Supplementary file 2 — Additional file 2: Supplementary Table 2. Characteristics of nulliparas by package of maternity care. [file 12884_2020_3199_MOESM2_ESM.docx]

Supplementary Table 2. Characteristics of nulliparas by package of maternity care.

|  |  | Total | Public | Semi-private | Private |
| --- | --- | --- | --- | --- | --- |
|  | *n* | *n*=29376 | *n*=22395 | *n*=3283 | *n*=3698 |
| Age (years; mean, SD) | 29376 | 29.4 (5.8) | 28.1 (5.6) | 32.5 (3.8) | 34.4 (4.2) |
| Age <35 years (%) | 23936 | 81.5 | 87.2 | 72.1 | 55.2 |
| Age 35-39 years (%) | 4462 | 15.2 | 10.8 | 24.4 | 33.5 |
| Age ≥ 40 years (%) | 978 | 3.3 | 2.0 | 3.5 | 11.3 |
| Elective CS (%) | 2119 | 7.2 | 4.9 | 6.7 | 21.7 |
| Emergency CS (%) | 6494 | 22.1 | 21.8 | 23.9 | 22.3 |
| Vaginal delivery (%) | 20754 | 70.7 | 73.3 | 69.4 | 56.0 |
| Married/Civil Partnership (%) | 16804 | 57.2 | 48.7 | 80.7 | 88.0 |
| Irish-born (%) | 20185 | 68.9 | 62.5 | 88.5 | 89.8 |
| Infertility treatment (%) | 1684 | 5.7 | 3.4 | 7.0 | 18.8 |
| Planned pregnancy (%) | 18746 | 63.9 | 59.5 | 80.4 | 75.5 |
| BMI (median, IQR) | 29188 | 23.9 (5.5) | 23.9 (5.0) | 24.2 (5.0) | 23.7 (4.6) |
| Underweight (%) | 928 | 3.2 | 3.1 | 1.3 | 4.9 |
| Normal weight (%) | 16764 | 57.1 | 56.4 | 57.5 | 60.5 |
| Overweight (%) | 7766 | 26.4 | 26.2 | 29.1 | 25.2 |
| Obesity (%) | 3918 | 13.3 | 14.2 | 12.1 | 9.4 |
| Professional/managerial employment (%) | 7887 | 27.1 | 18.3 | 46.9 | 62.1 |
| Unemployed (%) | 2392 | 8.2 | 10.6 | 0.9 | 0.7 |
| Current depression (%) | 381 | 1.3 | 1.5 | 0.7 | 0.5 |
| Current anxiety (%) | 1257 | 4.3 | 4.8 | 3.5 | 2.1 |
| Anxiolytics/antidepressants (%) | 456 | 1.6 | 1.6 | 1.0 | 1.5 |
| Smoked in pregnancy (%) | 3253 | 11.1 | 13.8 | 3.0 | 1.5 |
| Any alcohol use in pregnancy (%) | 391 | 1.3 | 1.2 | 1.9 | 1.4 |
| Illicit drugs in pregnancy (%) | 726 | 2.5 | 3.1 | 0.9 | 0.1 |
