# Supplementary material for: Caesarean section rates in women in the Republic of Ireland who chose to attend their obstetrician privately: a retrospective observational study
Source: BMC Pregnancy Childbirth. 2020 Sep 21;20:548. doi: 10.1186/s12884-020-03199-x (PMC7504647; doi:10.1186/s12884-020-03199-x)
Supplement: Supplementary file 3 — Additional file 3: Supplementary Table 3. Characteristics of multiparas by package of maternity care. [file 12884_2020_3199_MOESM3_ESM.docx]

Supplementary Table 3. Characteristics of multiparas by package of maternity care.

|  |  | Total | Public | Semi-private | Private |
| --- | --- | --- | --- | --- | --- |
|  | *n* | *n*=43890 | *n*=32677 | *n*=10.5 | *n*=6591 |
| Age (years; mean, SD) | 43890 | 32.6 (5.0) | 31.6 (5.1) | 34.9 (3.4) | 36.1 (3.4) |
| Age <35 years (%) | 26989 | 61.5 | 70.1 | 44.1 | 31.0 |
| Age 35-39 years (%) | 13907 | 31.7 | 24.8 | 43.8 | 54.1 |
| Age ≥ 40 years (%) | 2294 | 6.8 | 5.1 | 7.6 | 14.9 |
| Elective CS (%) | 8138 | 18.5 | 15.4 | 19.4 | 33.8 |
| Emergency CS (%) | 3786 | 8.6 | 8.9 | 7.1 | 8.2 |
| Vaginal delivery (%) | 31954 | 72.8 | 75.7 | 73.5 | 58.0 |
| Married/Civil Partnership (%) | 30279 | 69.0 | 60.9 | 90.6 | 94.4 |
| Irish-born (%) | 31186 | 71.3 | 64.2 | 91.5 | 91.8 |
| Infertility treatment (%) | 965 | 2.2 | 1.1 | 2.8 | 7.0 |
| Planned pregnancy (%) | 29921 | 68.2 | 62.6 | 83.4 | 85.2 |
| BMI (median, IQR) | 43529 | 25.0 (6.3) | 25.2 (6.7) | 24.6 (5.1) | 24.0 (5.0) |
| Underweight (%) | 1003 | 2.3 | 2.0 | 0.8 | 4.7 |
| Normal weight (%) | 21069 | 48.0 | 45.5 | 52.4 | 57.1 |
| Overweight (%) | 13432 | 30.6 | 31.1 | 32.8 | 26.7 |
| Obesity (%) | 8386 | 19.1 | 21.4 | 13.9 | 11.4 |
| Professional/managerial employment (%) | 10833 | 24.8 | 15.8 | 40.7 | 58.3 |
| Unemployed (%) | 3167 | 7.3 | 9.6 | 0.9 | 0.4 |
| Current depression (%) | 811 | 1.8 | 2.3 | 0.7 | 0.5 |
| Current anxiety (%) | 1447 | 3.3 | 3.7 | 2.6 | 1.8 |
| Anxiolytics/antidepressants (%) | 1077 | 2.5 | 2.8 | 1.4 | 1.5 |
| Smoked in pregnancy (%) | 5956 | 13.6 | 17.5 | 3.3 | 1.2 |
| Any alcohol use in pregnancy (%) | 723 | 1.6 | 1.7 | 2.2 | 1.2 |
| Illicit drugs in pregnancy (%) | 432 | 1.0 | 1.3 | 0.2 | 0.1 |
