# Supplementary material for: Caesarean section rates in women in the Republic of Ireland who chose to attend their obstetrician privately: a retrospective observational study
Source: BMC Pregnancy Childbirth. 2020 Sep 21;20:548. doi: 10.1186/s12884-020-03199-x (PMC7504647; doi:10.1186/s12884-020-03199-x)
Supplement: Supplementary file 4 — Additional file 4: Supplementary Table 4. Changes in mode of delivery between pregnancies by package of maternity care in second pregnancy (n = 11,990). [file 12884_2020_3199_MOESM4_ESM.docx]

Supplementary Table 4. Changes in mode of delivery between pregnancies by package of maternity care in second pregnancy (n=11,990).

|  |  | | Package of care second pregnancy | | | | | | Total  *n*=11,990 | |
| --- | --- | --- | --- | --- | --- | --- | --- | --- | --- | --- |
|  |  |  | Private  *n*=1908 | | Semi-private  *n*=1578 | | Public  *n*=8504 | |  |  |
| Total VBAC | n | 77 | | 110 | | 456 | | 643 | |  |
|  | % | 4.0% | | 7.0% | | 5.4% | | 5.4% | |  |
| VBAC (Elective CS first pregnancy) | n | 15 | | 25 | | 87 | | 127 | |  |
|  | % | 0.8% | | 1.6% | | 1.0% | | 1.1% | |  |
| VBAC (Emergency CS first pregnancy) | n | 62 | | 85 | | 369 | | 516 | |  |
|  | % | 3.2% | | 5.4% | | 4.3% | | 4.3% | |  |
| Vaginal both | n | 991 | | 1039 | | 5990 | | 8020 | |  |
|  | % | 51.9% | | 65.8% | | 70.4% | | 66.9% | |  |
| Elective both | n | 259 | | 56 | | 198 | | 513 | |  |
|  | % | 13.6% | | 3.5% | | 2.3% | | 4.3% | |  |
| Emergency CS to Elective CS | n | 325 | | 215 | | 917 | | 1457 | |  |
|  | % | 17.0% | | 13.6% | | 10.8% | | 12.2% | |  |
| Vaginal to elective CS | n | 103 | | 45 | | 244 | | 392 | |  |
|  | % | 5.4% | | 2.9% | | 2.9% | | 3.3% | |  |
| Elective CS to Emergency CS | n | 31 | | 18 | | 58 | | 107 | |  |
|  | % | 1.6% | | 1.1% | | 0.7% | | 0.9% | |  |
| Emergency both | n | 67 | | 57 | | 370 | | 494 | |  |
|  | % | 3.5% | | 3.6% | | 4.4% | | 4.1% | |  |
| Vaginal to emergency CS | n | 55 | | 38 | | 271 | | 364 | |  |
|  | % | 2.9% | | 2.4% | | 3.2% | | 3.0% | |  |
