# Supplementary material for: Caesarean section rates in women in the Republic of Ireland who chose to attend their obstetrician privately: a retrospective observational study
Source: BMC Pregnancy Childbirth. 2020 Sep 21;20:548. doi: 10.1186/s12884-020-03199-x (PMC7504647; doi:10.1186/s12884-020-03199-x)
Supplement: Supplementary file 5 — Additional file 5: Supplementary Table 5. Factors associated with changes in model of care between private and public. [file 12884_2020_3199_MOESM5_ESM.docx]

Supplementary Table 5. Factors associated with changes in model of care between private and public.

| Factor | *n* | Private to public | Public to private |
| --- | --- | --- | --- |
|  |  | *n* = 144 | *n* = 155 |
| Age 2^nd^ pregnancy |  |  |  |
| >30 years | 8565 | Reference | Reference |
| <30 years | 3426 | 2.8 (1.3-5.9)^b^ | 0.4 (0.3-0.7)^b^ |
| BMI category 2^nd^ pregnancy |  |  |  |
| Underweight | 241 | 0.9 (0.3-3.4) | 0.4 (0.1-3.0) |
| Normal weight | 6295 | Reference | Reference |
| Overweight | 3542 | 0.9 (0.6-1.4) | 0.8 (0.5-1.2) |
| Obesity | 1913 | 1.2 (0.7-2.1) | 0.9 (0.5-1.4) |
| Occupation 2^nd^ pregnancy |  |  |  |
| Professional/managerial | 4019 | Reference | Reference |
| Other non-manual/skilled manual | 4814 | 1.5 (1.1-2.3)^c^ | 0.3 (0.2-0.5)^a^ |
| Semi-skilled/unskilled manual | 628 | 4.5 (1.2-16.8)^c^ | 0.1 (0.1-0.4)^a^ |
| Unemployed | 748 | 9.3 (2.0-43.2)^b^ | 0.4 (0.2-1.0) |
| Homemaker | 1782 | 0.6 (0.2-1.7) | 0.3 (0.1-0.5)^a^ |
| Marital status 2^nd^ pregnancy* |  |  |  |
| Married | 8388 | Reference | Reference |
| Single | 3511 | 1.4 (0.7-2.6) | 0.4 (0.2-0.7)^a^ |
| Separated/Divorced | 84 | - | 0.8 (0.1-5.9) |
| Pregnancy intention^ |  |  |  |
| Planned | 9195 | Reference | Reference |
| Unplanned | 2429 | 4.9 (2.6-9.1)^a^ | 0.8 (0.4-1.4) |
| Infertility treatment | 364 | 0.4 (0.2-1.0) | 2.7 (1.4-5.5)^b^ |
| Smoking^ |  |  |  |
| Never | 6476 | Reference | Reference |
| Ex-smoker | 4537 | 1.4 (0.9-2.1) | 1.0 (0.7-1.5) |
| Current smoker | 975 | 2.7 (0.6-11.9) | 0.5 (0.1-1.6) |
| Postnatal depression |  |  |  |
| No | 11368 | Reference | Reference |
| Yes | 623 | 1.1 (0.3-3.6) | 0.8 (0.3-1.8) |
| Antidepressants/Anxiolytics^~^ |  |  |  |
| No | 11778 | Reference | Reference |
| Yes | 208 | 1.6 (0.5-5.3) | 0.5 (0.1-3.4) |
| Caesarean section 1^st^ pregnancy |  |  |  |
| No | 8777 | Reference | Reference |
| Elective CS | 747 | 0.7 (0.4-1.3) | 1.2 (0.6-2.5) |
| Emergency CS | 2467 | 0.8 (0.5-1.2) | 1.7 (1.2-2.5)^b^ |
| Previous miscarriage |  |  |  |
| No | 8453 | Reference | Reference |
| Yes | 3538 | 1.1 (0.7-1.6) | 1.6 (1.2-2.2)^b^ |

Overall reference group: Private in both pregnancies *n*=1633.

All variables included in the tables were mutually adjusted for in the regression analyses.

^a^p<0.001; ^b^p<0.01; ^c^p<0.05.

Missing data **n*=8, ^^^*n*=3, ^~^*n*=5.
